# Gestational trophoblastic neoplasm and women living with HIV and/or AIDS

**DOI:** 10.4102/sajhivmed.v16i1.344

**Published:** 2015-07-03

**Authors:** Pieter Barnardt, Martha Relling

**Affiliations:** 1 Department of Medical Imaging and Clinical Oncology, Division of Radiation Oncology, University of Stellenbosch, Tygerberg Campus, South Africa

## Abstract

The 2011 World Health Organization global report on HIV and/or AIDS estimated that sub-Saharan Africa comprised 67% of the global HIV burden, with a current estimate of 5.9 million cases in South Africa. Since the introduction of antiretroviral therapy, there has been an increase in the incidence of non-AIDS-defining cancers. Gestational trophoblastic neoplasm (GTN) is a rare pregnancy-related disorder with an incidence ranging from 0.12–0.7/1000 pregnancies in Western nations. The overall cure rate is about 90%. Response to treatment for GTN is generally favourable; but the sequelae of HIV and/or AIDS, the resultant low CD4 counts, comorbidities, poor performance status and the extent of metastatic disease in patients receiving chemotherapy, compromise the prognosis and survival.

## Introduction

Infection with the human immune deficiency virus (HIV) in sub-Saharan Africa affected an estimated 22.5 million people, of whom 5.9 million were in South Africa, however, in the recent 2014 UNAIDS progress report, for the first time since 2009, a 17% decline was reported in the rate of newly diagnosed HIV cases for women of reproductive age, living in sub-Saharan Africa.^1^ May et al. found that, between 1996 and 2008, the life expectancy of people receiving antiretroviral therapy (ART) had increased by an average of 15 years.^2^ People with HIV infection live longer as a result of ART, and consequently are at risk of developing illnesses associated with ageing, chronic illnesses and malignancies.

Kaposi’s sarcoma (KS), non-Hodgkin’s lymphoma (NHL) and cervical cancer are the most observed AIDS-defining cancers. In the era of ART, a significant decrease in the incidence of AIDS-defining illnesses has been observed, whilst non-AIDS cancers (NADCs) are on the increase. An estimated 30% – 40% of HIV-infected patients are likely to develop a cancer during the duration of their disease.^3^ An increased incidence was observed for anal, lung, certain head and neck cancers, and hepatocellular carcinoma, as well as Hodgkin’s disease, whilst no increased risk was observed for breast, prostate and colorectal cancer in the HIV-positive cohort. No clearly established association between HIV and/or AIDS and gestational trophoblastic neoplasm (GTN) exists. Data from the Swiss HIV Cohort Study showed that 19% of all cohort deaths in the ART area were attributable to NADCs.^4^

GTN is a rare pregnancy-related disorder that derives from placental tissue and is clinically diagnosed if the B-HCG level fails to decrease or normalise in women when a normal pregnancy is excluded, and collectively includes: invasive mole, choriocarcinoma and placental site trophoblastic tumour (PSTT) that can lead to death if left untreated. Potential risk factors for the development of GTN include a history of a previous molar pregnancy, partial mole (0.5% risk) and a complete mole (15% risk), maternal age and a post-evacuation, persistent raised B-HCG level. Management includes careful dilatation and curettage (D&C), and the need for systemic treatment is guided by the International Federation of Gynecology and Obstetrics (FIGO)/WHO score that predicts if either single agent or combination chemotherapy is required. GTN is now one of the most curable solid tumours, with cure rates of more than 90% even in the presence of metastatic disease.^5,6^ The occurrence of malignancy amongst people living with HIV and/or AIDS represents a management challenge. We present here two choriocarcinoma case studies of women with HIV and/or AIDS.

## Ethics approval

The Human Research Ethics Committee (HREC) of Stellenbosch University approved the present report.

## Case presentation

### Case 1

**FIGURE 1:**
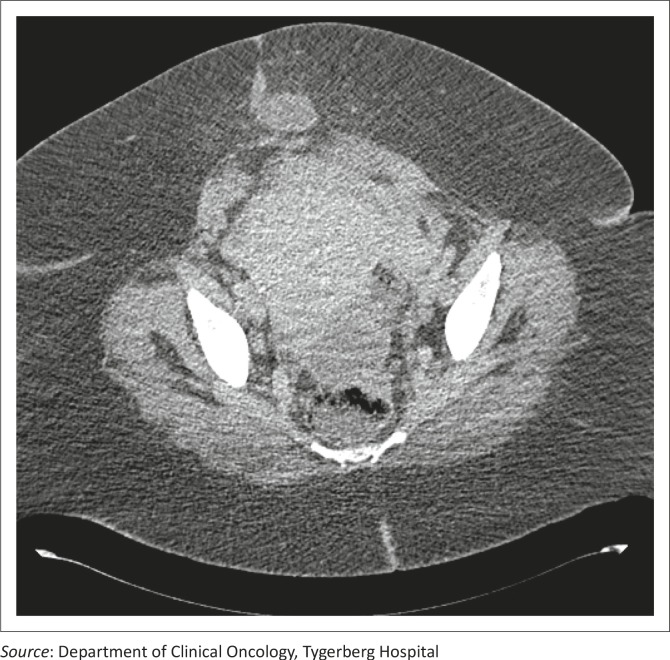
Computer tomographic scan of the abdomen demonstrating a grossly enlarged uterus and tumour mass extending through the anterior abdominal wall to the subcutaneous tissue.

A 33-year-old woman (gravida 5, para 2, miscarriage 3) presented post-evacuation for a miscarriage with persistent vaginal bleeding and a raised B-HCG value. She was living with HIV and/or AIDS and had been established on ART for one year. Clinical examination confirmed an abdominal uterine mass of 22 weeks’ gestation with a foul-smelling vaginal discharge. Staging examinations included a computed tomography (CT) scan of the lungs, abdomen and pelvis and confirmed several bilateral lung metastases and an extensive tumour that involved the uterus and extended into the anterior abdominal wall (Figure 1). Abnormal laboratory studies revealed haemoglobin 4 g% (12.5 g% – 15 g%),a serum B-HCG level of 115 544 IU/L (<5 IU/L), and CD4 count of 290 cells/μL, (500 cells/μL – 2010 cells/μL). She was diagnosed as having a FIGO Stage III:12 high-risk choriocarcinoma.

Management included continuation with ART (tenofovir, efavirenz and lamivudine), intravenous antibiotics (piper­acillin and amikacin), and low-dose chemotherapy with the alternating regime of methotrexate 50 mg alternative days (D1, 3, 5, 7) with leucovorin rescue. She completed one cycle without any adverse effects. However, a week post-chemotherapy she developed a neutropaenic fever. Abnormal laboratory studies then confirmed serum-creatinine 10.7 mmol/L (2.1 mmol/L – 7.1 mmol/L), urea 151 μmol/L (49 μmol/L – 90 μmol/L), and a pancytopaenia: white blood cells 0.13 × 10^9^/L (4.00–10.00 ×10^9^/L), haemoglobin 6.6 g%, and platelets 15 × 10^9^/L (178–400 ×10^9^/L). A follow-up CD4 count decreased to 29 cells/μL and the B-HCG value decreased to 50 296 IU/L. Supportive management included granulocyte-stimulating factor (G-CSF) and intravenous antibiotics according to the sensitivity of a positive blood culture (*Klebsiella pneumonia*). A vaginal pus swab cultured Candida species.

The outcome for the patient was unfortunate as she died owing to severe neutropaenic sepsis from a resistant Klebsiella and Candida sepsis that resulted in acute renal failure and severe immune suppression.

### Case 2

**FIGURE 2:**
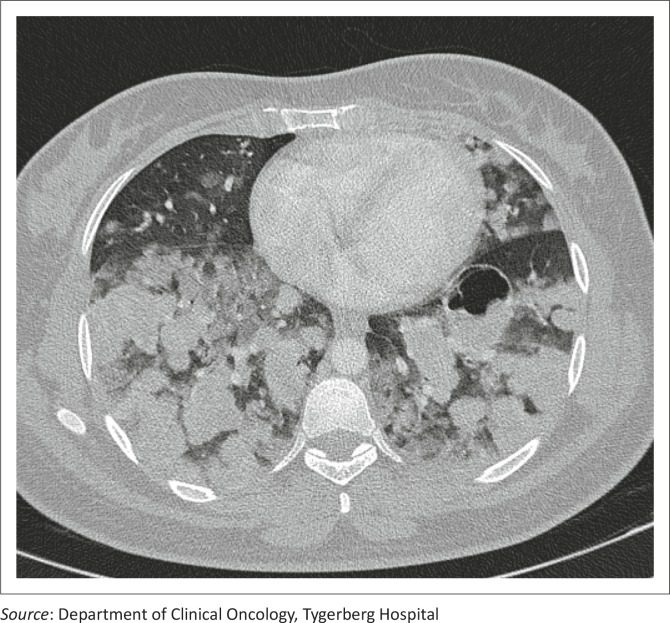
Computer tomographic scan of the lung demonstrating bilateral diffuse soft-tissue nodules in keeping with metastatic disease. Note the large cavitating lesion in the left lower lobe, found in metastases from choriocarcinoma and resulting from tumour haemorrhage.

A 20-year-old woman (gravida 2, para 0, miscarriage 1) presented with a history of haemoptysis, grade IV dyspnoea and a raised B-HCG level. She was known to have had a previous molar pregnancy, diagnosed three years previously. On clinical examination, symptoms and signs of thyrotoxicosis were present. Staging examinations included a lung CT that confirmed numerous bilateral, diffuse soft-tissue nodules throughout both lung fields in keeping with metastatic disease (Figure 2). Abnormal laboratory studies revealed a B-HCG value >898 400 IU/L (<5 IU/L), T4: 47 pmol/L (10.3 pmol/L – 21.9 pmol/L), and a CD4 count of 200 cells/μL (500 cells/μL – 2010 cells/μL); a HIV enzyme-linked immunosorbent assay (ELISA) test was positive. Direct microscopy for acid-fast bacilli was negative. She was diagnosed with a FIGO Stage III:15 high-risk choriocarcinoma in a newly diagnosed HIV-positive patient.

Management included commencement with ART (tenofovir, efavirenz and lamivudine) with the addition of trimetroprim-sulfamethoxazole for *Pneumocystis jiroveci* prophylaxis (PCP) as her CD4+ count was ≤200 cells/μL, intravenous antibiotic (clindamycin) and acute thyrotoxicosis therapy consisting of carbimazole and propranolol. Low-dose chemotherapy with methotrexate 50 mg alternated with leucovorin rescue on days 1, 3, 5 and 7 was initiated. However, she required admission into a high-care facility when spontaneous breathing became problematic and a continuous positive airway pressure (CPAP) support system was needed. A standard blood culture confirmed Gram-positive cocci and a coagulase negative staphylococcal organism was isolated sensitive to vancomycin. A urine specimen cultured positive for *Candida albicans*.

When severe oxygen decompensation occurred, she was admitted to the ICU facility and intubation was necessitated. She died secondary to poor performance status, severe immune suppression, metastatic choriocarcinoma and a Staphylococcus and Candida sepsis when she developed acute respiratory distress syndrome (ARDS).

## Discussion

Malignancy tends to occur at a younger age in people living with HIV and/or AIDS, and presents with atypical presentations, widespread metastatic disease, and aggressive tumour behaviour. In patients with immunodeficiency, physiological principles for the development of malignancy exist: (1) the lack of autoimmune surveillance, (2) an imbalance between cellular differentiation and proliferation and (3) a repeat antigenic stimulation by an oncogenic virus leads to the emergence and proliferation of abnormal cells. Amongst HIV-infected individuals, a 30% – 40% increase in the incidence of NADCs has been observed, and this contributes to morbidity and mortality in HIV-infected patients, now that survival is prolonged with the use of ART.^3,7^

Established chemotherapy regimens have resulted in a favourable response to GTN, and more than 90% of cases will be cured. Choriocarcinoma is generally associated with pregnancy but an estimated 25% occur after miscarriage, 25% after term pregnancy and the rest after a molar pregnancy. Choriocarcinoma is an aggressive form of GTN owing to its rapid growth and metastatic potential. In women, a high index of suspicion is needed to make a diagnosis based on an unexplained high B-HCG level in the presence of metastases in the lung, liver or brain. A high HCG level can also cause thyrotoxicosis. Delay in diagnosis with delay in starting chemotherapy treatment is a common cause of early death in patients with metastasis.^6^

Using the WHO/FIGO scoring system, patients are grouped into low- and high-risk categories. High-risk patients require combination chemotherapy as they are unlikely to be cured with a single agent. In high-risk patients, 50% of deaths occur within the first four weeks of initial treatment. Early deaths are attributed to respiratory compromise, haemorrhage secondary to a heavy tumour burden within the thorax, and rapid tumour destruction associated with full-dose chemotherapy treatment. Intubation in patients with very poor respiratory function should be avoided as far as possible as high ventilator pressures might trigger fatal intrapulmonary haemorrhage owing to friable tumour vasculature; and hence the introduction of low-dose therapy in the initial treatment of high-risk patients to gradually reduce tumour volume and significantly reduce the risk of haemorrhage to minimise the risk of early death.^8,9^

Because of extensive comorbidities, low-CD4 counts, poor performance status and the extent of metastatic disease, both our patients were offered initial low-dose chemotherapy to debulk tumour load. In the first case study, our patient developed post-chemotherapy neutropaenic sepsis. The outcome for her resulted in death secondary to a poor performance, severe immune suppression (CD4 count decrease from 290 cells/μL to 29 cells/μL), neutropaenic sepsis and extensive metastatic choriocarcinoma that resulted in acute renal failure. In the second case, the patient experienced severe oxygen desaturation owing to extensive lung metastases that necessitated intubation and ICU admission. The outcome for her was dismal as she died secondary to a poor performance status, severe immune suppression, sepsis and extensive metastatic choriocarcinoma when she developed ARDS.

Both our patients experienced severe immune suppression, had extensive metastatic disease and developed associated post-chemotherapy neutropaenia that increased their susceptibility to developing severe and fatal infections. Our cases also highlight previous findings of reported studies on GTN and HIV that confirm an associated poor outcome related to chemotherapy in women with HIV and/or AIDS if they present with low CD4 counts, have a poor performance status and an associated poor tolerance to chemotherapy treatment.^10,11^

## Conclusion

Response to GTN is generally favourable with cure rates in excess of 90%. However, in patients with advanced immune suppression, medical management of non-AIDS-defining cancers can be compromised owing to their extent and aggressive tumour behaviour with associated treatment-related complications.
